# Untangling the Roots of HPV Vaccine Acceptance in West Virginia: How Hesitancy and Misinformation Shape Parental Decisions

**DOI:** 10.3390/vaccines14070608

**Published:** 2026-07-10

**Authors:** Jeanine P. D. Guidry, Linnea I. Laestadius, Yil Engbersen-Severijns, Carrie A. Miller, Michael P. Stevens, Candace W. Burton, Kellie E. Carlyle, Janina-Marie Huss, Kathryn Moffett, Paul B. Perrin

**Affiliations:** 1 Department of Communication and Cognition, Tilburg University, 5037 AB Tilburg, The Netherlands; 2 Joseph J. Zilber College of Public Health, University of Wisconsin-Milwaukee, Milwaukee, WI 53205, USA; llaestad@uwm.edu; 3 Department of Health Psychology, Open University, 6419 AT Heerlen, The Netherlands; yil.severijns@ou.nl; 4 Department of Family Medicine and Population Health, School of Medicine, Virginia Commonwealth University, Richmond, VA 23219, USA; carrie.a.miller@vcuhealth.org; 5 Department of Internal Medicine, Division of Infectious Diseases, School of Medicine, West Virginia University, Morgantown, WV 26506, USA; mike.stevens@wvumedicine.org; 6 School of Nursing, York University, Toronto, ON M3J 1P3, Canada; cwburton@yorku.ca; 7 Department of Social and Behavioral Sciences, School of Public Health, Virginia Commonwealth University, Richmond, VA 23219, USA; kecarlyle@vcu.edu; 8 West Virginia University School of Medicine, West Virginia University Health System, Morgantown, WV 26506, USA; janina-marie.huss@wvumedicine.org; 9 Department of Pediatrics, School of Medicine, West Virginia University, Morgantown, WV 26506, USA; kmoffett@hsc.wvu.edu; 10 School of Data Science, Department of Psychology, University of Virginia, Charlottesville, VA 22904, USA; perrin@virginia.edu

**Keywords:** HPV vaccine, misinformation, vaccine hesitancy, West Virginia, parents

## Abstract

Background: West Virginia is of particular concern regarding HPV vaccine hesitancy, currently ranking 45th among U.S. states in HPV vaccine uptake. General vaccine hesitancy and HPV- and HPV vaccine-specific misinformation, both associated with lower vaccination, have increased in recent years, complicating efforts to identify key drivers of HPV vaccine acceptance and effective intervention targets. Methods: This study used a Qualtrics survey of *n* = 330 parents of children aged 0–14 in West Virginia. Measures included parental HPV vaccine acceptance for their youngest child (reflecting either completed vaccination or intention to vaccinate), general vaccine hesitancy measured using the Vaccine Hesitancy Scale (VHS), and endorsement of nine common HPV- and HPV vaccine-specific misinformation statements. Misinformation items were summed and demonstrated good internal reliability (Cronbach’s α = 0.868). Results: Hierarchical logistic regression analyses indicated significant racial/ethnic differences across all model steps, with non-White parents less likely to report HPV vaccine acceptance for their child (*p* < 0.001). In Step 2, lower levels of general vaccine hesitancy predicted higher HPV vaccine acceptance (*p* < 0.001). In the final model, higher endorsement of HPV- and HPV vaccine-specific misinformation was associated with lower HPV vaccine acceptance (*p* = 0.035). Conclusions: These findings underscore the independent and additive influence of general vaccine hesitancy and HPV- and HPV vaccine-specific misinformation on parental HPV vaccine acceptance in West Virginia. Community-engaged public health strategies should prioritize strengthening vaccine confidence and directly addressing HPV- and HPV vaccine-specific misinformation, particularly among underserved populations.

## 1. Introduction

Human papillomaviruses (HPV) contribute substantially to the global cancer burden, accounting for an estimated 37,800 cancer diagnoses annually in the United States (U.S.) and approximately 690,000 worldwide [1,2]. To reduce the burden of HPV-associated disease, prophylactic vaccines targeting the most common oncogenic HPV types were introduced in 2006 [3]. Evidence from population-based studies and systematic reviews demonstrates that HPV vaccination is associated with marked declines in HPV infections, genital warts, and precancerous cervical lesions, particularly when administered prior to exposure to the virus [4]. In the United States, the Advisory Committee on Immunization Practices (ACIP) recommends routine HPV vaccination at ages 11–12, although vaccination may begin as early as age 9. Catch-up vaccination is further recommended for individuals up to age 26 who have not previously completed the vaccination series [5]. Although HPV vaccination is widely recommended, coverage remains suboptimal both nationally and internationally [6,7], falling short of vaccination targets established by Healthy People 2030 and the World Health Organization [8,9]. Furthermore, important disparities in HPV vaccine uptake persist across racial/ethnic groups and between rural and non-rural populations [10,11].

Appalachia, a predominantly rural region of the eastern United States (U.S.) associated with persistent health and socioeconomic disparities, has consistently reported both lower HPV vaccine initiation rates and higher cervical cancer rates compared to other regions in the U.S. [12,13]. West Virginia, the only state entirely located in Appalachia, reported cervical cancer rates of 9.0/100,000 for the female population per year between the years of 2010–2014, compared to the national rate of 7.5/100,000 [14]. Studies of HPV vaccine coverage demonstrate lower uptake rates for populations living in Appalachia compared to the rest of the U.S. [15], with just under 50% uptake in West Virginia as of 2023 [16].

One important factor that may contribute to persistently low HPV vaccination rates is vaccine hesitancy. Vaccine hesitancy, defined as a delay in acceptance or refusal of safe vaccines despite the availability of vaccination services, is considered one of the ten main global health threats by the World Health Organization (WHO) [17], and has been significantly increasing in the 21st century. Recent studies have shown that vaccine misinformation, defined as statements about vaccination that are contrary to the best available evidence as defined by public health authorities [18,19], is associated with increasing numbers of infectious disease cases, including those caused by HPV [20,21]. The spread of vaccine misinformation has accelerated dramatically through the explosive growth of social media platforms (e.g., Instagram, TikTok) in the past decade, which allow such messages to reach much wider audiences, including ones that do not actively seek scientifically validated information [22,23,24]. Individuals who delay or refuse vaccines are significantly more likely to search for vaccine information online [25,26], and parents who rely on the Internet for vaccination information are significantly less likely to trust healthcare providers and health authorities as reliable sources [27]. Concerningly, anti-vaccine discourse tends to have greater reach and persuasive impact than pro-vaccine messages [28], and vaccine hesitancy has been directly linked to the spread of misinformation on social media [29].

Frequent false claims about vaccines in general include the myth that they are manufactured using aborted fetal stem cells, a particularly important concern among conservative Christians, relevant given the relatively high proportion of conservative Christians in West Virginia [30,31], and the myth that vaccines cause autism [26]. Within the broader spectrum of vaccines and their associated misinformation beliefs, HPV vaccine hesitancy takes a somewhat unique place, shaped by issues related to sexual behavior and parental concerns about their children’s potential early sexual activity [32]. This makes it critical to discern to what extent low uptake of the vaccine is driven by concerns specific to the HPV vaccine versus broader concerns about vaccines. Common false claims about the HPV vaccine include that it increases sexual promiscuity in young adolescents, causes infertility, leads to severe adverse effects (e.g., autoimmune diseases, neurological diseases, death), that boys do not need the vaccine [33,34], and conflating HPV and HIV [35,36].

Although vaccination intention and completed vaccine uptake are conceptually distinct, HPV vaccination decision-making among parents often unfolds over time and is influenced by children’s age-based eligibility for vaccination. Examining both completed vaccination and intention may therefore provide insight into parental HPV vaccine acceptance across different stages of the decision-making process. West Virginia’s combination of elevated HPV-related cancer burden and relatively low HPV vaccination coverage makes understanding parental vaccination decision-making particularly important. Identifying the factors that influence acceptance of the HPV vaccine may help inform more effective communication and intervention strategies. The goal of this study was to investigate how overall vaccine hesitancy and HPV-specific vaccine misinformation endorsement are associated with HPV vaccination acceptance, including both completed vaccination and future vaccination intentions, among West Virginia parents. We hypothesized that both (1) higher overall vaccine hesitancy and (2) higher levels of HPV misinformation endorsement would be associated with lower HPV vaccine acceptance.

## 2. Materials and Methods

Participants (*N* = 330) were recruited through Qualtrics, a managed online panel service of individuals who have agreed to participate in survey research. Recruitment ran from 2 March to 16 April 2025. Potential participants were screened for eligibility based on the study inclusion criteria prior to accessing the survey. Eligible participants completed the survey online and received compensation through the panel provider according to standard panel procedures. To be eligible, participants had to be West Virginia residents 18 or older and have at least one child between the ages of 0–14 living in their home. This age range was selected to capture both current HPV vaccination decisions and future vaccination intentions among parents whose children would become eligible for HPV vaccination during adolescence. After receiving an invitation, interested panelists entered the Qualtrics study environment. They were presented with an information letter and after giving informed consent, were asked to complete a 3-question screener to determine eligibility. Participants who did not consent were not allowed to continue to the screening questions, and participants who did not meet eligibility criteria were directed to the end of the survey. Participants who completed the survey received a point-based reward from Qualtrics. This study received ethical approval by the Institutional Review Board of Tilburg University, a large research university in the Netherlands (Approval: REDC2024.60).

The survey included the following measures:

HPV vaccination behaviors. HPV vaccine acceptance was assessed based on a similar measure [37] using the following single item: “Please indicate your intention to vaccinate your youngest child with the HPV vaccine when they are recommended based on their age” that had four response options: “My child has already received the HPV vaccine,” “I am planning to get my child the HPV vaccine as soon as possible,” “I am uncertain about whether I will get my child the HPV vaccine,” and “I will not get my child the HPV vaccine.” Because HPV vaccination eligibility depends on child age, responses were operationalized as a binary measure of HPV vaccine acceptance. Parents reporting that their child had already received the HPV vaccine or that they intended to vaccinate their child were categorized as accepting, while parents who were uncertain or unwilling to vaccinate were categorized as hesitant. This approach was used to capture parental HPV vaccine acceptance across different stages of the vaccination decision-making process.

Parent and child demographic characteristics. Parent age, child age, parent sex, child sex, race/ethnicity, education, and rurality were collected.

Overall vaccine hesitancy. Overall vaccine hesitancy was measured using the Vaccine Hesitancy Scale (VHS) [38], consisting of nine items, of which seven were reverse-coded (e.g., “Childhood vaccines are important for my child’s health,” which was reverse-coded; and “New vaccines carry more risks than older vaccines”). The mean of the nine items on the scale was used in the calculations, with higher scores indicating higher levels of vaccine hesitancy.

Cronbach’s alpha for the scale was 0.907.

Endorsement of HPV- and HPV vaccine-specific misinformation. Endorsement of misinformation was measured using the following question: “In this question, you will read several statements about the HPV vaccine. To your best ability, without checking for information with others or online, answer whether you believe these statements are true or not true” and answer options “Definitely not true,” “Likely not true,” “Not sure if true or not true,” “Likely true,” and “Definitely true.” Nine statements were presented, e.g., “The HPV vaccine may cause severe adverse effects, including death” and “Getting the HPV vaccine will result in adolescents being more sexually active.” Cronbach’s alpha for the items was 0.868, and the scores for the items were therefore added and the mean calculated with a higher score indicating higher level of endorsement of misinformation.

## 3. Statistical Methods

Using SPSS 30.0, descriptive statistics and univariate analyses were conducted to explore differences in vaccine acceptance by parental characteristics. Hierarchical logistic regression was then used to examine the incremental contribution of demographic characteristics, vaccine hesitancy, and endorsement of HPV-related misinformation to predicting HPV vaccination behaviors. Variables were entered in a theory-driven sequence. Demographic characteristics were entered first because they represent relatively stable background factors associated with vaccination behaviors in prior research. General vaccine hesitancy, measured by the Vaccine Hesitancy Scale (VHS), was entered second because it reflects a broad predisposition toward vaccination. Finally, endorsement of HPV-related misinformation statements were entered in the third step because they represent proximal beliefs and perceptions hypothesized to explain additional variance in vaccine acceptance beyond demographics and general vaccine attitudes.

The effects of the independent variables at each step were reported as odds ratios (ORs) with 95% confidence intervals. Model fit and the proportion of variance explained were assessed using the Nagelkerke *R*^2^ at each step. To address the conceptual distinction between completed HPV vaccination and future vaccination intention, a sensitivity analysis was conducted excluding parents whose child had already received the HPV vaccine. Hierarchical logistic regression analyses were repeated among parents of non-vaccinated children, comparing parents who intended to vaccinate their child with those who were uncertain or unwilling to vaccinate, using the same predictor variables and model specification as the primary analysis.

No missing data were present in the dataset.

## 4. Results

Among the 330 survey participants, 65.2% were female and 34.8% male; 43.0% responded about a female child and 57.0% about a male child. The mean age of parents was 40.4 years (*SD* = 9.88), and of their youngest child 7.7 years (*SD* = 4.24). Most participants identified as White (87.9%), with 12.1% identifying as non-White. In terms of education, 23.9% held a bachelor’s or graduate degree, while 76.1% had less than a bachelor’s degree. Residence was reported as rural (38.2%), suburban (34.5%), or urban (27.3%) (Table 1). Of the total sample, 77.9% of parents reported their child had already received at least one dose of the HPV vaccine or they were planning to get their child the HPV vaccine (designated as “Vaccinated”), and 22.1% said they were undecided or definitely not going to get their child vaccinated (designated as “Not vaccinated”). The mean overall vaccine hesitancy score was 2.00 on a five-point scale (*SD* = 0.73). The mean and standard deviation for the nine items on the misinformation endorsement score was 2.72 (*SD* = 1.14) on a five-point scale.

### 4.1. Bivariate Analyses

Demographically, chi-square tests indicated that people of color were less likely to report their child was vaccinated or that they intended to have the child vaccinated compared to white respondents (*p* < 0.001). Parent sex, child sex, education, and rurality did not show any significant difference between vaccinated and not vaccinated children. T-tests showed that neither parent age nor child age was significantly associated with HPV vaccination acceptance. However, t-tests did show that those with a higher VHS score, signifying a higher level of vaccine hesitancy, were less likely to report HPV vaccination acceptance for their youngest child, as did a higher endorsement of HPV- and HPV vaccine-specific misinformation (both *p* < 0.001) (Table 1).

### 4.2. Multivariate Analyses

**Overall analysis.** For the first step of the logistic regression predicting HPV vaccine acceptance, sociodemographic variables (parent and child age and sex, race/ethnicity, education, and rurality) were entered; X^2^(8) = 18.44, *p* = 0.018. Compared to people of color, white respondents were more likely to report HPV vaccine acceptance for their child (OR = 3.984; *p* < 0.001); none of the other demographic variables were significant (Table 2). The model explained 8.3% of the variance in HPV vaccine acceptance (Nagelkerke *R*^2^ = 0.083).

In Step 2, the Vaccine Hesitancy Scale score was added as a predictor, X^2^(9) = 57.040, *p* < 0.001. Race/ethnicity was still significant and in the same direction (OR = 3.929; *p* < 0.001). In addition, higher overall vaccine hesitancy was associated with lower HPV vaccine acceptance (OR = 0.327; *p <* 0.001) (see Table 2). Demographics and vaccine hesitancy accounted for 24.3% of the variation in HPV vaccine acceptance.

For the third step, endorsement of misinformation about HPV vaccination was added as a predictor, X^2^(10) = 64.563, *p* < 0.001. In this step, white respondents were still more likely to report HPV vaccine acceptance for their child (OR = 3.776; *p* < 0.001). None of the other demographics were significant, though vaccine hesitancy remained significant (OR = 0.369; *p* < 0.001). Participants more strongly endorsing HPV vaccine misinformation were less likely to report HPV vaccine acceptance for their child (OR = 0.647; *p* = 0.007). The final model explained 27.2% of the variance in HPV acceptance (Nagelkerke *R*^2^ = 0.272), suggesting that demographics, overall vaccine hesitancy, and HPV- and HPV vaccine-related misinformation endorsement jointly contributed to parental HPV vaccine acceptance; see Table 2.

**Sensitivity analysis.** To assess the robustness of findings when distinguishing future vaccination intention from completed vaccination behavior, a sensitivity analysis was conducted among parents whose child had not yet received the HPV vaccine (*n* = 197). For the first step of the logistic regression predicting HPV vaccination intention, sociodemographic variables (parent and child age and sex, race/ethnicity, education, and rurality) were entered; X^2^(8) = 9.504, *p* = 0.302. Compared to people of color, white respondents were more likely to report HPV vaccination intention for their child (OR = 2.837; *p* = 0.012); none of the other demographic variables were significant (Table 3). The model explained 6.4% (Nagelkerke *R*^2^) of the variance in HPV vaccination intention.

In Step 2, the Vaccine Hesitancy Scale score was added as a predictor; X^2^(9) = 37.583, *p* < 0.001. Race/ethnicity was still significant and in the same direction (OR = 2.693; *p* = 0.027). In addition, higher overall vaccine hesitancy predicted lower HPV vaccination intention for their child (OR = 0.306; *p* < 0.001) (see Table 3). Demographics and vaccine hesitancy accounted for 23.7% of the variation in HPV vaccination intention.

For the third step, endorsement of misinformation about HPV vaccinations was added as a predictor; X^2^(10) = 42.606, *p* < 0.001. In this step, white respondents were still more likely to report HPV vaccination intention for their child (OR = 2.513; *p* = 0.043). None of the other demographics were significant. General vaccine hesitancy remained significant (OR = 0.369; *p* < 0.001). Participants more strongly endorsing HPV- and HPV vaccine-specific misinformation were less likely to report HPV vaccination intention for their child (OR = 0.668; *p* = 0.027). The *R*^2^ value of 0.266 suggested that among West Virginia parents, demographics, overall vaccine hesitancy, and endorsement of HPV vaccine-related misinformation together explained 26.6% of the variation in HPV vaccination intention (Table 3). Overall, findings from the sensitivity analysis were substantively similar to those observed in the primary analysis.

Based on these results, both hypotheses were supported: Higher overall vaccine hesitancy and higher HPV misinformation endorsement were both significantly associated with lower HPV vaccine acceptance. These associations remained substantively similar in sensitivity analyses restricted to parents of non-vaccinated children, supporting lower intention to vaccinate. This pattern suggests that vaccine hesitancy and misinformation endorsement may be relevant across multiple stages of parental decision-making, including both uncertainty about and unwillingness toward HPV vaccination.

## 5. Discussion

This study examined factors associated with HPV vaccine acceptance among parents of children ages 0–14 in West Virginia, focusing on sociodemographic characteristics, overall vaccine hesitancy, and endorsement of HPV vaccine-related misinformation. Several important findings emerged, including the relatively high proportion of parents who reported that their youngest child had either already been vaccinated against HPV or that they planned to do so. At 77.9%, the HPV vaccine acceptance rate in this sample—reflecting either completed vaccination or intention to vaccinate—was notably higher than current estimates for HPV vaccination completion among children and adolescents, particularly in rural and Appalachian areas [16,39]. While encouraging, this figure should be interpreted with some caution, as it may reflect data collection factors such as the use of a panel sample or social desirability bias, rather than broader statewide trends. Because this measure combined completed vaccination behavior with future vaccination intention, findings should not be interpreted as reflecting HPV vaccine uptake alone. Although intention does not always translate into behavior, sensitivity analyses restricted to parents of non-vaccinated children yielded substantively similar findings, suggesting robustness of the observed associations.

Overall vaccine hesitancy and endorsement of HPV vaccine-related misinformation were both significant and independent predictors of lower HPV vaccine acceptance, even after adjusting for demographic variables. Importantly, these associations remained substantively similar in sensitivity analyses restricted to parents of children who had not yet received the HPV vaccine, suggesting that findings were robust when examining future vaccination intention separately from completed vaccination behavior. This finding aligns with prior studies showing that parental vaccine hesitancy remains a critical barrier to HPV vaccine acceptance across diverse populations [40,41]. Importantly, these results underscore that overall vaccine hesitancy may not simply be due to demographic differences, but also reflects deeper concerns related to safety and necessity.

Endorsement of HPV- and HPV vaccine-related misinformation was also significantly and independently associated with lower vaccine acceptance. This suggests that, beyond general hesitancy, exposure to and belief in false or misleading information specific to the HPV vaccine may have a distinct impact on parental decision-making. In light of the increasing influence of social media and politicized discourse on vaccine attitudes, these findings reinforce the need for targeted misinformation response strategies and clear, evidence-based public health messaging [42]. Countering misinformation through trusted messengers should be a central priority for vaccine promotion in West Virginia and elsewhere.

Among the demographic variables examined, only race/ethnicity was a consistent and significant predictor of HPV vaccine acceptance. While many studies have shown that racial/ethnic minority populations often have higher HPV vaccination initiation than their White counterparts, there are lower completion rates for the vaccine series [43]. In this study, across all three steps of the regression analysis, White parents were significantly more likely than parents of color to report HPV vaccine acceptance for their child. This disparity persisted even after accounting for vaccine hesitancy and misinformation endorsement and remained directionally similar in sensitivity analyses. This finding differs from much of the U.S. literature and may reflect the unique demographic and healthcare context of West Virginia. Prior research suggests that HPV vaccination in Appalachian populations is shaped by region-specific factors including healthcare access, transportation challenges, preventive care utilization, and cultural attitudes toward healthcare and vaccination [15,44]. In addition, provider recommendation is one of the strongest predictors of HPV vaccine uptake, and previous studies have found that Appalachian healthcare providers may be less likely to recommend HPV vaccination than providers in non-Appalachian settings [45]. Thus, racial/ethnic differences observed in the present study may reflect the intersection of race/ethnicity with broader rural and Appalachian healthcare disparities rather than race/ethnicity alone.

While limited sample sizes prevented subgroup analyses among non-White participants, the finding raises important equity concerns, particularly since people of color disproportionally carry the burden of poor HPV-related outcomes such as cervical cancer [46]. Structural and systemic factors such as historical and contemporary medical mistrust and disparities in healthcare access likely contribute to this difference [47,48]. These results highlight the need for HPV vaccine acceptance interventions that specifically engage and build trust with parents of color.

In contrast, other demographic variables (parent and child sex, parental education level, rurality, and age) were not significantly associated with HPV vaccine acceptance. This lack of association may reflect the specific cultural and informational environment of West Virginia, where vaccine attitudes may be shaped more by community norms, historical experiences, and interpersonal trust than by standard sociodemographic markers. Although more than one-third of respondents reported living in rural areas, rurality itself was not predictive of vaccine acceptance in the primary model or vaccination intention in sensitivity analyses, suggesting that within this sample, rural residence may be less strongly associated with HPV vaccine acceptance than broader factors such as beliefs and misinformation endorsement [49].

These findings carry important implications for public health practice. The continued influence of misinformation and hesitancy indicates that major gaps remain in encouraging HPV vaccine acceptance among parents of eligible children and adolescents. Public health efforts in West Virginia should prioritize both: (1) directly addressing HPV vaccine misinformation and tailoring it to community concerns, as well as encouraging training for healthcare providers to respond effectively to HPV vaccination concerns, and (2) continuing to build trust in vaccines more broadly and in healthcare providers who recommend and administer them, as well as supporting outreach to communities that have historically experienced healthcare exclusion or mistreatment [50,51].

## 6. Strengths, Limitations and Future Directions

The primary strength of this study is its inclusion of parental endorsement of HPV vaccine misinformation as a factor influencing HPV vaccine acceptance for their children. This extends existing research on vaccine hesitancy by capturing how belief in HPV vaccine misinformation may further shape parental decision-making.

Despite its contributions, this study has several limitations. Its cross-sectional nature limits causal inference and prevents determination of the direction of the observed associations. While vaccine hesitancy and misinformation endorsement were associated with lower HPV vaccine acceptance, reverse causation is also possible, such that parents who are less accepting of HPV vaccination may be more likely to endorse vaccine-related misinformation or report higher levels of general vaccine hesitancy. In addition, data were self-reported, which introduces the possibility of bias. Individuals who are willing to complete an online research survey are more likely to have a higher level of education, as well as more openness to science [52]. As a result, some of the most vaccine-hesitant parents may have been less likely to participate in this study. This may partially explain the relatively high rate of HPV vaccine acceptance observed in the sample and limits generalizability to the broader West Virginia population. In addition, while the sample captured important variation within West Virginia, the results may not generalize to other regions or racial/ethnic groups, particularly given the relatively small number of non-White participants. Future research should prioritize recruitment strategies to enhance racial and ethnic diversity within West Virginia and increase participation among parents who may be more vaccine-hesitant, particularly regarding HPV vaccination. Alternative recruitment strategies, including in-person outreach through schools, clinics, faith communities, and local health departments, may help address these challenges.

Furthermore, the primary outcome combined completed HPV vaccination and future vaccination intention to account for age-based HPV vaccine eligibility among children ages 0–14. As such, the findings should be interpreted as reflecting HPV vaccine acceptance across different stages of parental decision-making rather than determinants of vaccination behavior alone. Although sensitivity analyses among parents of non-vaccinated children yielded substantively similar findings, intention and completed behavior remain conceptually distinct constructs and should be examined separately in future longitudinal research. In addition, because many children in the sample had not yet reached the recommended age for HPV vaccination, parental responses may reflect anticipated rather than immediate vaccination decisions; however, such intentions remain important given the role of early attitudes in shaping future vaccine uptake. Longitudinal and experimental study designs may also better clarify causal relationships between vaccine hesitancy, misinformation endorsement, and HPV vaccination behavior over time.

As with all online panel surveys, the findings should be interpreted in light of potential selection bias. Individuals who participate in online research panels may differ from the broader population in ways that could influence vaccine-related attitudes and behaviors. In addition, individuals without reliable internet access are less likely to be represented in the sample.

Finally, although the nine misinformation items reflect commonly propagated HPV vaccine misinformation beliefs, they do not represent a comprehensive list. Moreover, although the items demonstrated good internal consistency, they were not part of a validated scale. Future studies should endeavor to develop and validate a more comprehensive, psychometrically sound measure of endorsed HPV vaccine misinformation to improve reliability and comparability across studies.

## 7. Conclusions

This study contributes to the growing body of literature identifying key drivers of HPV vaccine acceptance among parents by highlighting the significant roles of general vaccine hesitancy and HPV-specific misinformation in the state of West Virginia. Despite relatively encouraging HPV vaccine acceptance rates in this sample, the study’s findings point to ongoing and critical barriers, particularly among parents of color, that must be addressed to reduce HPV-related cancer incidence. The independent and additive effects of vaccine hesitancy and misinformation endorsement suggest that public health strategies will need to go beyond generic pro-vaccine messaging. Efforts should additionally focus on building trust in vaccines, directly countering misinformation, and delivering tailored, culturally sensitive outreach to the communities most affected. Future research should prioritize longitudinal and representative studies to better capture the complexity of parental decision-making around HPV vaccination. Without sustained, evidence-based interventions at the intersection of science communication, trust in vaccinations, and health equity, disparities in HPV vaccine coverage are likely to persist, leaving preventable cancers to continue exacting an uneven toll.

## Figures and Tables

**Table 1:** Descriptives of the sample (*N* = 330).

Parent Characteristics	Overall % (*n =* 330)	*p*-Value
Parent sex		0.322
Female	65.2% (*n* = 215)	
Male	34.8% (*n* = 115)	
Parent age, years		0.967
Mean, SD	40.4, 9.88	
Child sex		0.488
Female	43.0% (*n* = 142)	
Male	57.0% (*n* = 188)	
Child age, years		0.217
Mean, SD	7.7, 4.24	
Race/ethnicity		<0.001 *
White	87.9% (*n* = 290)	
People of Color	12.1% (*n* = 40)	
Education		0.442
Less than bachelor’s	76.1% (*n* = 251)	
Bachelor’s or higher	23.9% (*n* = 79)	
Rurality		0.693
Rural	38.2% (*n* = 126)	
Suburban	34.5% (*n* = 114)	
Urban	27.3% (*n* = 90)	
Vaccine Hesitancy Scale		<0.001 *
Mean, SD	2.0, 0.73	
Misinformation endorsement		<0.001 *
Mean, SD	2.74, 1.09	

* *p* < 0.05.

**Table 2:** Hierarchical logistic regression predicting HPV vaccine acceptance.

Variable	OR	95%CI	*p*-Value	OR	95%CI	*p*-Value	OR	95%CI	*p*-Value
Parent age	0.986	0.96, 1.02	0.349	0.986	0.96, 1.02	0.401	0.987	0.96, 1.02	0.441
Child age	1.024	0.95, 1.10	0.519	1.01	0.94, 1.09	0.797	1.007	0.93, 1.09	0.898
Parent sex: Female (Ref: Male)	1.201	0.66, 2.18	0.545	1.308	0.69, 2.49	0.427	1.200	0.62, 2.31	0.708
Child sex	0.934	0.54, 1.62	0.808	1.064	0.59, 1.93	0.838	1.096	0.60, 2.00	0.696
Education: Bachelor’s (Ref: Less than bachelor’s)	1.638	0.80, 3.35	0.177	1.679	0.77, 3.65	0.191	1.706	0.77, 3.77	0.173
Race: White (Ref: POC)	3.984	1.91, 8.29	<0.001 *	3.929	1.79, 8.64	<0.001 *	3.776	1.70, 8.39	0.001 *
Rurality: Urban (Ref: Rural)	0.646	0.32, 1.32	0.231	0.641	0.30, 1.40	0.262	0.651	0.30, 1.42	0.296
Rurality: Suburban (Ref: Rural)	0.844	0.44, 1.61	0.608	0.691	0.34, 1.40	0.307	0.691	0.34, 1.41	0.320
Vaccine hesitancy				0.327	0.21, 0.52	<0.001 *	0.396	0.25, 0.64	<0.001 *
Misinformation endorsement							0.647	0.47, 0.89	0.007 *

* *p* < 0.05.

**Table 3:** Hierarchical logistic regression predicting HPV vaccine uptake intent ONLY.

Variable	OR	95%CI	*p*-Value	OR	95%CI	*p*-Value	OR	95%CI	*p*-Value
Parent age	0.998	0.97, 1.03	0.913	1.001	0.97, 1.04	0.957	1.00	0.97, 1.04	0.954
Child age	0.974	0.90, 1.06	0.527	0.953	0.87, 1.04	0.287	0.957	0.88, 1.05	0.343
Parent sex: Female (Ref: Male)	1.323	0.68, 2.59	0.414	1.486	0.72, 3.07	0.284	1.244	0.59, 2.64	0.569
Child sex	1.287	0.69, 2.40	0.428	1.387	0.71, 2.72	0.341	1.457	0.73, 2.90	0.282
Education: Bachelor’s (Ref: Less than bachelor’s)	1.736	0.78, 3.87	0.177	1.905	0.79, 4.60	0.153	1.853	0.75, 4.60	0.183
Race: White (Ref: POC)	2.837	1.26, 6.40	0.012 *	2.693	1.12, 6.47	0.027 *	2.513	1.04, 6.13	0.043 *
Rurality: Urban (Ref: Rural)	0.824	0.38, 1.80	0.625	0.793	0.34, 1.86	0.594	00.830	0.35, 1.98	0.674
Rurality: Suburban (Ref: Rural)	0.882	0.43, 1.82	0.732	0.708	0.32, 1.56	0.393	0.733	0.33, 1.64	0.449
Vaccine hesitancy				0.306	0.21, 0.46	<0.001 *	0.369	0.25, 0.64	<0.001 *
Misinformation endorsement							0.668	0.47, 0.95	0.027 *

* *p <* 0.05.

## Data Availability

The dataset for this study is available from the corresponding author upon request.

## Abbreviations

The following abbreviations are used in this manuscript:

HPV	Human Papillomaviruses
ACIP	Advisory Committee on Immunization Practices
U.S.	United States
